# Scientific research progress of COVID‐19/SARS‐CoV‐2 in the first five months

**DOI:** 10.1111/jcmm.15364

**Published:** 2020-05-07

**Authors:** Hua Li, Zhe Liu, Junbo Ge

**Affiliations:** ^1^ Department of Cardiology Shanghai Institute of Cardiovascular Diseases Shanghai Xuhui District Central Hospital and Zhongshan‐Xuhui Hospital Zhongshan Hospital Fudan University Shanghai China; ^2^ Department of Medicine BestNovo (Beijing) Medical Technology Co., Ltd Beijing China

## Abstract

A cluster of pneumonia (COVID‐19) cases have been found in Wuhan China in late December, 2019, and subsequently, a novel coronavirus with a positive stranded RNA was identified to be the aetiological virus (severe acute respiratory syndrome coronavirus 2, SARS‐CoV‐2), which has a phylogenetic similarity to severe acute respiratory syndrome coronavirus (SARS‐CoV). SARS‐CoV‐2 transmits mainly through droplets and close contact and the elder or people with chronic diseases are high‐risk population. People affected by SARS‐CoV‐2 can be asymptomatic, which brings about more difficulties to control the transmission. COVID‐19 has become pandemic rapidly after onset, and so far the infected people have been above 2 000 000 and more than 130 000 died worldwide according to COVID‐19 situation dashboard of World Health Organization (https://covid19.who.int). Here, we summarized the current known knowledge regarding epidemiological, pathogenesis, pathology, clinical features, comorbidities and treatment of COVID‐19/ SARS‐CoV‐2 as reference for the prevention and control COVID‐19.

## 1 BACKGROUND

In late December 2019, a cluster of pneumonia (COVID‐19) cases with unidentified causes have been found in Wuhan, Hubei Province, China. It is related to a positive stranded RNA virus (severe acute respiratory syndrome coronavirus 2, SARS‐CoV‐2), which has a phylogenetic similarity to severe acute respiratory syndrome coronavirus (SARS‐CoV).^1^ From the beginning, COVID‐19 was reported to be epidemiologically linked to the Huanan Seafood Wholesale Market, where there was sale of local fish and live wild animals.^2^ The subsequent evidence of clinician infection suggests that SARS‐CoV‐2 can transmit from human to human.^3^ Massive alveolar damage and progressive respiratory failure may lead to death in severe cases, and the counts of lymphocyte, monocyte, leucocyte, infection‐related biomarkers, inflammatory cytokines and T cells are also changed in severe patients.^2^, ^4^

Many diagnosis and treatment strategies have been taken to prevent the spread of SARS‐CoV‐2 and isolation is the most effective way. Detection of SARS‐CoV‐2 nucleic acid or specific IgM and IgG in serum has become a convenient way to identify COVID‐19. For hospitalized patients, drug treatment such as alpha interferon, lopinavir/ritonavir, ribavirin, chloroquine phosphate and arbidol, and convalescent plasma therapy may be potential options. Convalescent plasma therapy is mainly used for the severe and critical cases. In this article, we aim to describe the epidemiological, pathogenesis, pathology, clinical features, comorbidities and treatment of COVID‐19/SARS‐CoV‐2.

## 2 EPIDEMIOLOGY

So far, the COVID‐19 patients of nine countries have surpassed 50 000 and they are American, Spain, Italy, Germany, France, The United Kingdom, China, Iran and Turkey in a descending order. The number of confirmed cases and deaths of COVID‐19 was higher than SARS‐CoV (more than 8000 confirmed cases and 800 deaths worldwide) and MERS‐CoV (2494 confirmed cases and 858 deaths worldwide).^5^ In a study of 99 COVID‐19 cases, nearly half of patients (49) were clustered and had exposure history.^6^ According to a survey conducted by Chinese Centers for Disease Control and Prevention on more than 40,000 COVID‐19 patients, about 56% of the patients were men and the median age was 59 years with 87% 30‐79 years of age, 3% 80 years or older and 2% under 20 years old.^7^, ^8^ The overall case fatality rate (CFR) was 2.3%, in which the CFR of the elderly and patients with pre‐existing comorbid conditions was higher. The CFR of over 70‐year‐old and over 80‐year‐old (including 80 years old) was around 50.8% and 14.8% of the total number of deaths, respectively. No deaths occurred in the group aged 9 years and younger.^7^ The incubation period of COVID‐19 was 1‐14 days with mostly 3‐7 days, and the maximum incubation period could reach 24 days.^9^ A recent study constructed a model‐based analysis estimating the severity of COVID‐19 from the cases of 38 countries. The results showed that the mean duration from onset of symptoms to death and hospital discharge was 17.8 days (95% CI, 16.9‐19.2) and 24.7 days (22.9‐28.1), respectively. The case fatality ratio in China was 1.38% (1.23‐1.53), with substantially higher ratios in older age groups (6.4% [5.7‐7.2], ≥60 years) and up to 13.4% (11.2‐15.9) in those aged 80 years or older. Estimates of case fatality ratio from international cases stratified by age were consistent with those from China (4.5% [1.8‐11.1] in those aged ≥60 years [n = 151]).^10^

SARS‐CoV‐2 has strong transmission ability, and it has been occurred human‐to‐human transmission. The basic reproductive number (R_0_) of SARS‐CoV‐2 was estimated ~2.2 based on early patients and a subsequent study based on 75 815 individuals (from 31 December 2019 to 28 January 2020) estimated that R_0_ was 2.68.^5^, ^8^ Recent study from the Los Alamos National Laboratory has collected extensive individual case reports and designed mathematical modelling, which calculated the median R_0_ value as 5.7 (95% CI 3.8‐8.9).^11^ Therefore, the R_0_ of SARS‐CoV‐2 is rising with the increased number of confirmed cases and so far it has exceeded the R_0_ of MERS (R_0_ = 0.6) and SARS (R_0_ = 1).^12^ Scientists have predicted the trend of COVID‐19 development by studying its epidemic dynamics. It was indicated that Wuhan epidemic would peak around April 2020 and local epidemic across cities in mainland China would lag by 1‐2 weeks in a study.^5^ In another study, researchers estimated the epidemic peak would be on 17 February 2020 in China and an obvious rise could occur from 25 to 29 February 2020 overseas.^13^ Asymptomatic cases with COVID‐19 accounted for ~1% of the total number of patients and the viral load of asymptomatic patients was similar to that of symptomatic patients, which indicated that asymptomatic patients also had the potential to transmit virus.^7^, ^14^ SARS‐CoV‐2 is mainly transmitted by droplets, which invade the human body through contaminating the human conjunctival epithelium such as nose and eyes and cause infection.^15^ In addition to droplet transmission, faecal‐oral spread is also a potential mode of transmission.^16^ Although no studies have found COVID‐19 in newborns, specific antibodies have been detected in some newborns,^17^, ^18^ which indicated the possibility of transmission from the mother to her baby. Due to the particularity of droplet transmission, close contact activities, such as family clustering, usually increase the possibility of infection.^19^, ^20^ To block the viral transmission by isolation, wearing masks and other ways of reducing close contact are recommended.

## 3 PATHOGENESIS

### 3.1 Viral genome information and viral origin

**Figure 1:**
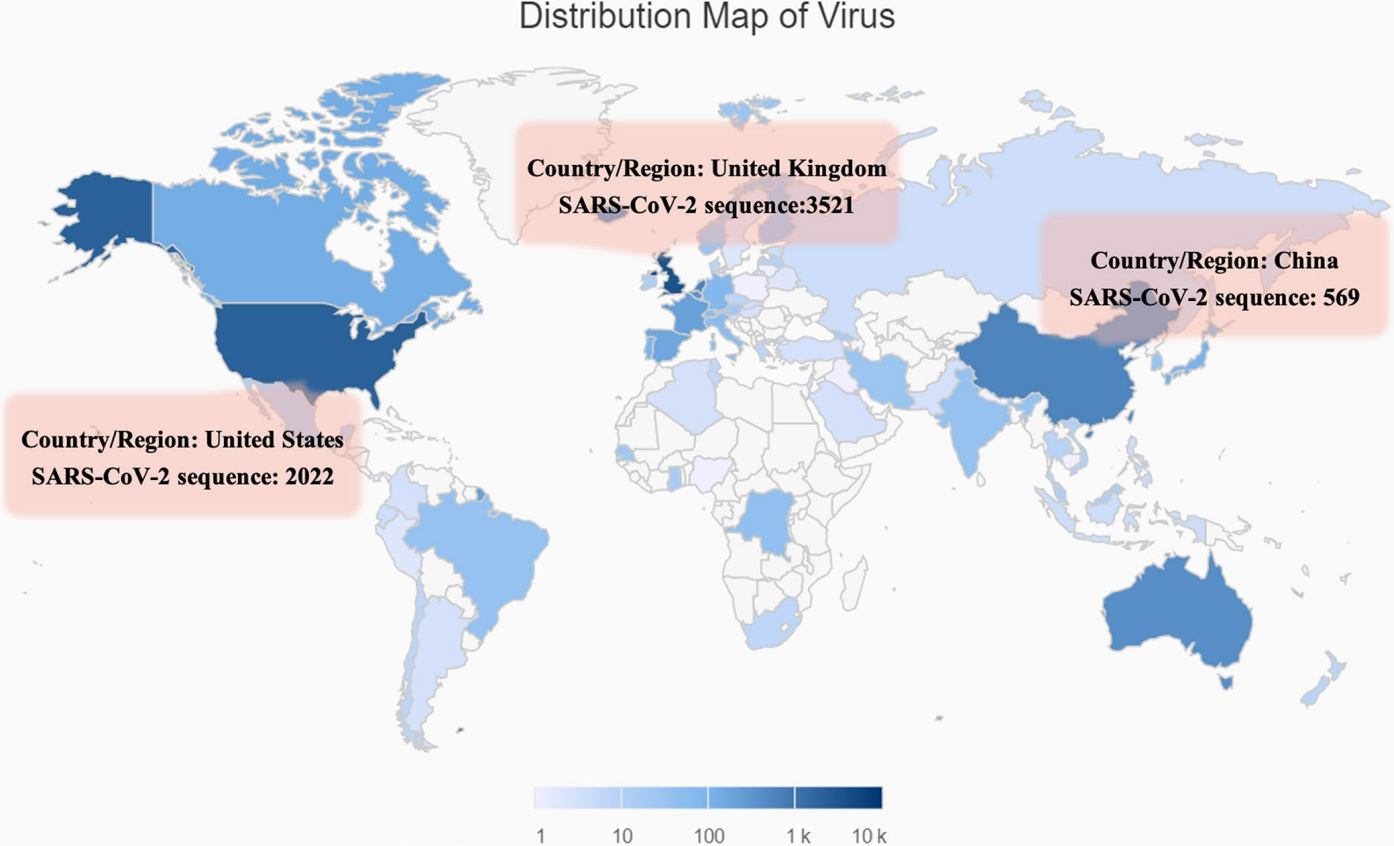
The distribution of virus submitted by different regions. In the world, China, United Kingdom and United States have uploaded 569, 3521 and 2022 virus sequences, respectively

Since COVID‐19 outbreak, the novel coronavirus SARS‐CoV‐2 causing the epidemic has been sequenced by worldwide scholars especially in China. A total of 9556 SARS‐CoV‐2 genome sequences have been uploaded in various global databases according to the data of the China National Center for Bioinformation 2019 Novel Coronavirus Resource (2019‐nCoVR) (Figure 1). Disclosure of the genome and amino acid sequence will contribute greatly to the viral classification and traceability, rapid nucleic acid detection of patients and understanding of the mechanism of viral infection and transmission. Especially, the clear viral sequence data could directly guide the selection of therapeutic agents and lay the foundation for subsequent vaccine and drug research and development.

SARS‐CoV‐2 is composed of at least 11 ORFs (Open Reading Frames) with the full length of 29 903 bp.^21^ Its genome is similar to SARS‐CoV with a gene order 5'‐ORF1ab‐S‐E‐M‐N‐3'. A large gene encoding a replicase (ORF1ab) is followed by 4 major structural protein‐coding genes: S (Spike protein), E (Envelope protein), M (Membrane protein) and N (Nucleocapsid protein). There are also at least 6 accessory ORFs.^21^, ^22^ SARS‐CoV‐2 belongs to the β coronavirus and its sequence significantly differs from that of SARS‐CoV and MERS‐CoV, which have caused epidemics previously.^1^ Sequence alignment displays that SARS‐CoV‐2 has 79% and 50% identity with SARS‐CoV and MERS‐CoV, respectively,^23^ whereas 96.3% identity with a bat's coronavirus.^24^ Phylogenetic analysis suggests that the closest relatives of SARS‐CoV‐2 are several viruses originated from bats, such as Bat‐CoV RaTG3, Bat SARSr CoV‐ZC45 and Bat SARSr CoV‐ZXC21. Therefore, bats are assumed to be the original host of SARS‐CoV‐2. However, there should be an intermediate host, who transfers the novel coronavirus to human because the sequence of SARS‐CoV‐2 and bat virus Bat‐CoV RaTG3 has minor difference.^23^, ^25^ The suspicious intermediate host has been suggested to be the pangolin by Chinese scientists on the basis of genetic analyses, which has not been confirmed until now with the release of new pangolin coronavirus genome studies.^26^ A recent study has found that the SARS‐COV‐2 was easy to infect cats and ferrets, but weakly infectious to dogs, pigs, chickens and ducks, which could be important for the traceability of the SARS‐COV‐2.^27^ Multiple sequence analysis studies have shown that the genome sequences of SARS‐CoV‐2 exhibit more than 99% sequence identity.^22^, ^23^, ^25^ As a typical RNA virus, the average substitution rate for coronavirus is 10^−4^ substitutions per year per site.^28^ The low heterogeneity among sequences suggests that all these SARS‐CoV‐2 originated from the same ancestor within a very short period.^23^ Despite the low heterogeneity, the number of viral genome variants will consistently increase with the continuous spread of COVID‐19. So far, 4007 variants have been identified and include single nucleotide polymorphism (SNP), deletion, indel and insertion with the mutant numbers of 3951, 82, 10 and 20, respectively (https://bigd.big.ac.cn/ncov). A deletion of eight amino acid has been reported in viral polyprotein 1ab (pp1ab) protein, encoded by ORF1ab gene of SARS‐CoV‐2. The pp1ab protein is composed of 16 mature non‐structural proteins (NSPs) and seldom reported to have mutations previously. This mutant was isolated from a Japanese COVID‐19 patient, who did not show critical symptoms.^29^ Domenico group described the presence of two mutations of SARS‐CoV‐2, affecting the NSP6 (amino acid position 3691) and ORF10 adjacent regions (amino acid position 9659), respectively, and both mutations could confer lower stability of the protein structures.^30^ Another two mutations of nt28144 in ORF8 and nt8782 in ORF1a of SARS‐CoV‐2 have been noticed to have higher mutation rate of 30.53% and 29.47%, respectively.^31^ A study analysed 103 SARS‐CoV‐2 genomes and also found nt28144 mutant in ORF8. They classified the SARS‐CoV‐2 virus as type L (28144T) and S (28144C) based on the different amino acid of leucine and serine in nt28144. S type was indicated to be the ancestral version although the L type (mutation rate ~70%) was more prevalent than the S type (mutation rate ~30%).^32^ L type was speculated to be more aggressive and contagious. By analysis of 160 complete human SARS‐CoV‐2 genomes, researchers from the University of Cambridge found three central variants distinguished by amino acid alteration, named A (8782T, 28144C), B (derived from A, 8782C, 28144T) and C (derived from B, 26144T), with A being the ancestral type according to the bat out‐group coronavirus.^33^ The A and C types were found mainly in Europeans and Americans, whereas the B type was the most common type in East Asia.

Currently, the reported mutation rate of SARS‐CoV‐2 is generally low and there is no evidence that viral recombination has occurred. Moreover, many countries have not published relevant virus genome information or only a small amount of genome information, which is obviously difficult to truly reflect the full picture of the SARS‐CoV‐2 mutation. Consequently, whether the variants of SARS‐CoV‐2 could affect its reproduction and transmission need further studies to clarify.

Recently, researchers have reported that they have successfully cloned and resurrected the novel coronaviruses using reverse genetics, a method of synthesizing viruses by cloning a known viral genome sequence. This method can provide researchers with infectious virus strains without valuable clinical samples and is an attractive alternative to obtain infectious virus strains.^34^

### 3.2 The receptor of SARS‐CoV‐2: Angiotensin converting enzyme II (ACE2)

The envelope spike (S) protein is crucial for determining host tropism and transmission capacity.^23^ Although the sequence identity of SARS‐CoV‐2 and SARS‐CoV is only 79%, their spike proteins have a highly similar three‐dimensional structure in the receptor‐binding domain (RBD),^35^, ^36^ which makes it possible that SARS‐CoV‐2, like SARS‐CoV, invades cells through the receptor ACE2. Viral infectivity studies using HeLa cells with and without ACE2 expression from humans, Chinese horseshoe bats, civets, pigs and mice reveal that SARS‐CoV‐2 is able to use all ACE2 except that of mouse as an entry receptor.^25^ To further understand the interaction of SARS‐CoV‐2 and ACE2 receptor, scientists from the United States and China separately analysed the cryo‐electronic microscope (cryo‐EM) structures of SARS‐CoV‐2 S protein^37^ and ACE2 receptor, as well as the three‐dimensional structure of S protein RBD and the full‐length protein complex of ACE2 receptor.^38^ Molecular modelling reveals that SARS‐CoV‐2 RBD interacts more strongly with ACE2 receptor than that of SARS‐CoV.^35^ Surface plasmon resonance analysis reveals that the affinity of human ACE2 protein binding to the novel coronavirus S protein is 10‐ to 20‐fold higher than that of ACE2 binding to SARS‐CoV S protein, which results in a stronger SARS‐CoV‐2 transmission capacity.^37^ These evidences strongly support that SARS‐CoV‐2 infects host cells by binding ACE2 receptor.

ACE2 belongs to a type I membrane protein expressed highly in lungs, oesophagus upper and stratified epithelial cells, intestine, absorptive enterocytes from ileum and colon, cholangiocytes, myocardial cells, kidney proximal tubule cells and testicle, which will be preferentially attacked.^39^, ^40^, ^41^ COVID‐19 transmission through faecal‐oral has been proved by evidences that diarrhoea and other alimentary system symptoms are the main manifestations of some patients^6^ and the stool samples of patients with diarrhoea are positive for SARS‐CoV‐2 nucleic acid test.^42^ The renal and testicle, highly expressing ACE2, may be potential to be invaded by SARS‐CoV‐2.^43^

**Figure 2:**
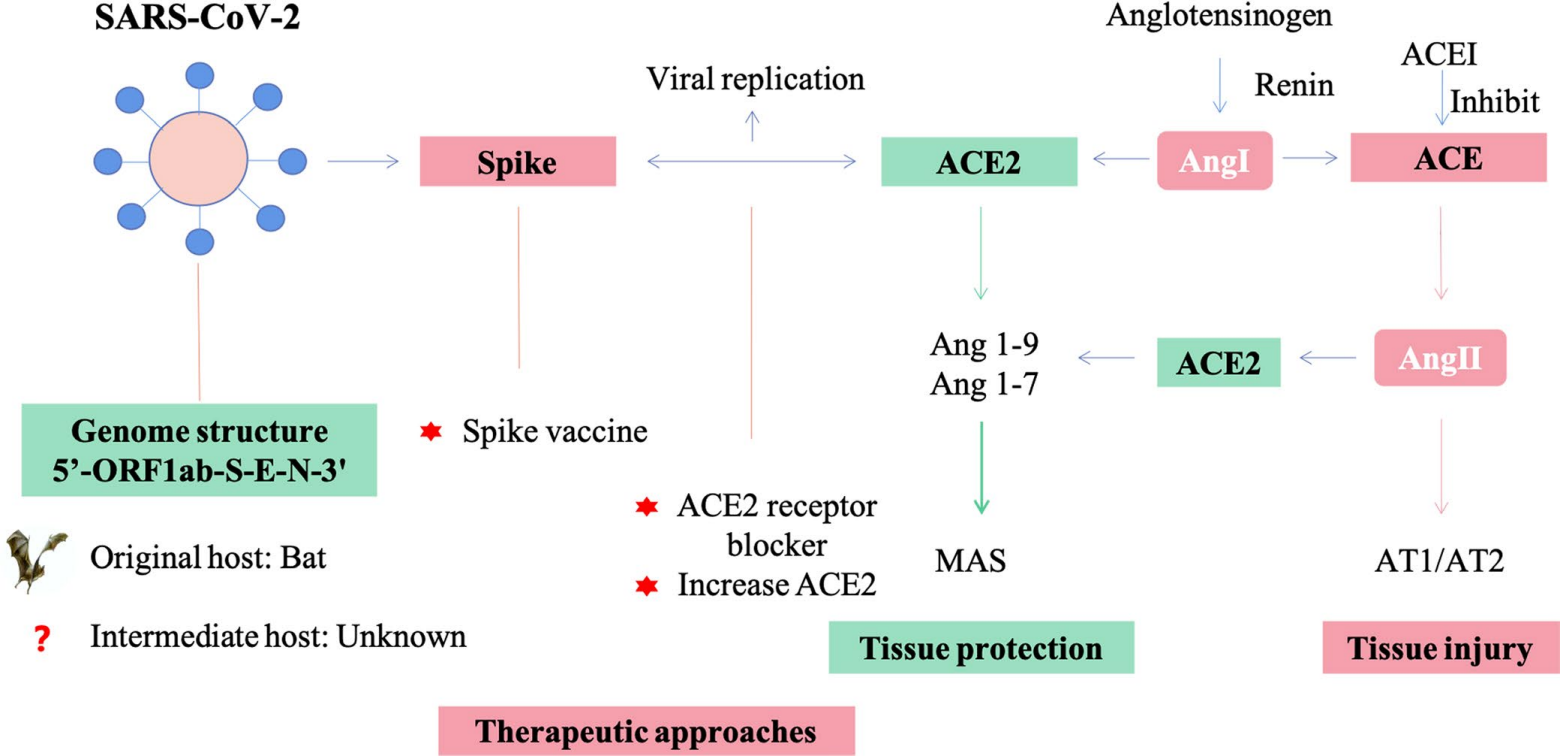
The mechanism of SARS‐CoV‐2 and ACE2 interaction based on renin‐angiotensin‐aldosterone system (RAAS) and potential therapeutic strategies in COVID‐19. SARS‐CoV‐2 invades cells via ACE2 receptor, which may lead to the down‐regulation of ACE2 expression. The down‐regulation of ACE2 expression could destroy the balance between ACE/ACE2 and lead to the tissue injury. Potential therapeutic approaches include a SARS‐CoV‐2 spike protein‐based vaccine and small‐molecule inhibitors to block the interaction between S protein and ACE2

The interaction between viral S protein and cell surface receptor ACE2 could activate the downstream renin‐angiotensin‐aldosterone system (RAAS) since ACE2 is one of the crucial components of RAAS (Figure 2). RAAS could be involved in the COVID‐19 pathogenesis and could balance the RAAS by increasing ACE2 or blocking the interaction between AngII, and AT1/AT2 could be a potential therapeutic target for COVID‐19.^44^

### 3.3 Cytokine storm

It was found that before death, the level of neutrophil count, D‐dimer, blood urea and creatinine levels of COVID‐19 patients continued to increase and the lymphocyte counts continued to decrease. These changes were related to cytokine storm, suggesting the activation of coagulation system, continuous inflammatory response and the occurrence of acute kidney injury, which could be involved into the pathogenesis of COVID‐19 exacerbation and explain the death of COVID‐19 patients.^45^

## 4 PATHOLOGY

Although greatly resembled pathological features of COVID‐19 with SARS and MERS coronavirus infection, COVID‐19 patients were found to have the unique pathological feature presenting as bilateral diffuse alveolar damage with cellular fibromyxoid exudates. Interstitial mononuclear inflammatory infiltrates dominated by lymphocytes were seen in both lungs, and multinucleated syncytial cells with atypical enlarged pneumocytes characterized by large nuclei, amphophilic granular cytoplasm and prominent nucleoli were identified in the intra‐alveolar spaces, showing viral cytopathic‐like changes.^46^

## 5 CLINICAL FEATURES

COVID‐19 is characterized by fever, weakness, dry cough, muscle soreness and dyspnoea. According to the study based on 1099 COVID‐19 hospitalized patients (926 non‐severe patients and 173 severe patients), fever was the most common symptom with 88.7% patients, followed by cough (67.8%), nausea or vomiting accounted for 5% of the total, 3.8% of the patients with diarrhoea and some patients with dizziness. Meanwhile, about 23.7% of the patients had hypertension or other underlying comorbidities.^9^, ^21^ A survey of 138 COVID‐19 hospitalized patients indicated that a considerable cases had comorbidities with 31.2% (43) hypertension, 10.1% (14) diabetes, 14.5% (20) cardiovascular disease and 7.2% (10) malignant.^45^

There are many COVID‐19 risk factors, and age, gender and blood type seem to be more important among them. In a retrospective, multicentre cohort study, older age is significantly correlated with COVID‐19, which is consistent with the higher incidence of older people. High Sequential Organ Failure Assessment (SOFA) score and D‐dimer >1 μg/L are also risk factors.^47^ Gender is another risk factor, and a lot of research have disclosed that higher prevalence of COVID‐19 in men than women.^48^ It may be related to sex‐based immunological or behavioural difference, such as smoking and so on.^49^ According to a research based on large population, A and O blood group displayed different association risks for COVID‐19. COVID‐19 patients with blood group A account for 37.75%, whereas the proportion is 32.16% in normal people. Respectively, the proportion of O group in COVID‐19 patients and normal people is 25.80% and 33.84%.^50^ This result is similar to the study focused on the relationship between ABO blood group and SARS‐CoV, which showed O blood group with a lower infection chance and anti‐A antibodies specifically inhibit the adhesion of SARS‐CoV S protein‐expressing cells to ACE2‐expressing cell lines.^51^, ^52^ These studies may contribute to the therapeutic strategy design of COVID‐19, but further studies need to be done to clarify the detailed mechanism.

In the early stage of COVID‐19, the total number of peripheral blood leucocytes was normal or decreased and the lymphocyte count was decreased. In some patients, liver enzyme, lactate dehydrogenase (LDH), muscle enzyme, aspartate transaminase level and myoglobin were increased.^53^ The numbers of helper T cells, suppressor T cells and regulatory T cells were significantly reduced in patients of severe COVID‐19, whereas the percentage of naïve T cells increased.^4^ Pneumonia is the typical symptom of COVID‐19, and the character of pulmonary image from high‐resolution computed tomography (HRCT) includes multiple small patchy shadows, interstitial changes, multiple ground glass opacities and infiltration in both lungs and so on.^9^, ^54^ In severe cases, pulmonary consolidation may occur whereas pleural effusion is rare.^54^, ^55^

## 6 COVID‐19 AND COMPLICATIONS

### 6.1 COVID‐19 and cardiovascular system

A study of 41 patients diagnosed with COVID‐19 showed that hypersensitive troponin I (hs‐cTnI) was increased substantially in five patients with the diagnosis of virus‐related cardiac injury. Four out of the five patients were admitted to the ICU, accounting for 31% of the total ICU patients.^2^ In a study of 138 COVID‐19 patients admitted to Zhongnan Hospital of Wuhan University, from 1 January to 28 January 2020, compared with non‐ICU patients, 36 severe patients receiving care in the ICU were more likely to have one of the complications such as cardiac injury and arrhythmia.^45^ Recently, a systematic review and meta‐analysis for 1813 COVID‐19 patients indicated that cardiovascular disease and hypertension were strongly predictive for both severe disease and ICU admission. However, they found that COPD was the most strongly predictive comorbidity for both severe disease (pOR 6.42, 95% CI 2.44‐16.9) and ICU admission (pOR 17.8, 95% CI 6.56‐48.2).

Studies have found that patients with the underlying cardiovascular diseases account for a large proportion of COVID‐19 patients. A study of the epidemiological and clinical characteristics of 99 COVID‐19 cases indicated that around 40 per cent had cardiovascular and cerebrovascular diseases.^6^ In one study of 138 patients, 58.3% patients with severe symptoms had hypertension.^45^ Another study analysed the clinical characteristics of 140 COVID‐19 patients and found that hypertension, diabetes and cardiovascular and cerebrovascular diseases were the most common underlying diseases in all patients.^56^ Therefore, it is important to improve the vigilance of patients with cardiovascular diseases.

Multiple evidences suggest that SARS‐CoV‐2 infects host cells by binding ACE2 receptor widely expressed in the cardiovascular system.^36^ Therefore, ACE2‐related signalling pathway may play a crucial role in cardiac injury. In addition, some scholars consider that there may be an imbalance between Th1 and Th2 responses in COVID‐19 patients and the resulting cytokine storm could also be another mechanism of cardiac injury.^2^ On the other hand, pneumonia caused by SARS‐CoV‐2 can lead to respiratory dysfunction and hypoxaemia, which can also bring about cardiomyocyte injury.^57^

### 6.2 COVID‐19 and tumour

A study of 1590 COVID‐19 patients indicated that patients with cancer had a higher risk of COVID‐19 (1% vs 0.29%) and a higher risk of severe events (a composite endpoint defined as the percentage of patients being admitted to the intensive care unit and requiring invasive ventilation or death) (39% vs 8%) than those without cancer.^58^ Current evidence remains insufficient to explain a conclusive association between cancer and COVID‐19. However, a recent communication in the Lancet refutes this viewpoint, arguing that current evidence is insufficient to explain the conclusive association between cancer and COVID‐19.^59^

### 6.3 COVID‐19 and kidney

Data from a clinical study involving 59 COVID‐19 patients showed that 19% (11/59) and 27% (16/59) of patients had an elevated level of plasma creatinine and urea nitrogen, respectively. All patients who underwent renal parenchymal CT had abnormal imaging of the kidney, mainly manifesting as low CT values of the renal parenchyma.^60^ A study of 1099 COVID‐19 patients found that the incidence of creatinine >133 micromol/L and acute kidney injury (AKI) were 4.3% and 2.9% in severe patients, respectively, whereas they were only 1% and 0.1%, respectively in non‐severe patients.^9^ Data from another study of 710 patients with COVID‐19 demonstrated that 44% of patients have proteinuria haematuria, 26.9% have haematuria and the prevalence of elevated serum creatinine and blood urea nitrogen were 15.5% and 14.1%, respectively on admission. During the study period, AKI occurred in 3.2% patients. Cox proportional hazard regression confirmed that elevated serum creatinine, elevated urea nitrogen, AKI, proteinuria and haematuria were the independent risk factors for in‐hospital death.^61^

Single‐cell RNA sequencing data revealed that ACE2 was highly expressed in kidney tubule cells, suggesting that the kidney was at high risk of coronavirus invasion.^62^ Moreover, compared with non‐ICU patients, ICU patients had higher plasma levels of IL‐2, IL‐7, IL‐10, GSCF, IP10, MCP1, MIP1A and TNF‐α, suggesting that the cytokine storm may be associated with disease severity.^2^

## 7 DETECTION METHODS

### 7.1 Nucleic acid detection

Detect SARS‐CoV‐2 nucleic acid by real‐time RT‐PCR via designing specific SARS‐CoV‐2 primers is crucial for COVID‐19 diagnosis. As above‐mentioned, SARS‐CoV‐2 has 79% and 50% identity with SARS‐CoV and MERS‐CoV, respectively^23^ whereas 96.3% identity with a bat's coronavirus.^24^ Compared the genomic sequences of SARS‐CoV‐2 with SARS‐CoV, regions coding ORF1ab gene, S gene, ORF7b, ORF8 genes and N genes are different.^63^ In S gene, SARS‐CoV‐2 shows lower GC% and higher AT% than SARS‐CoV and MERS. As for N gene, SARS‐CoV‐2 shows little difference in GC% and AT% compared with SARS‐CoV and MERS. However, the over‐biased codons are different. SARS‐CoV‐2 has 6 codons, including TTG, CTT, ATT, ACT, GCT and AGA, whereas SARS‐CoV and MERS show 4 and 5 over‐biased codons, respectively.^64^ Therefore, in consideration of the specific sequence of SARS‐CoV‐2, real‐time RT‐PCR detection of ORF1ab and N genes is recommended widely. If only one target can be detected due to the restriction of laboratory test kit, at least the ORF1ab region, the most conservative and specific for the new coronavirus, should be detected. Normally, the N gene is recommended as a screening assay and the ORF1ab assay as a confirmatory one. A result of N gene positive/ORF1ab negative should be regarded as indeterminate and the case is recommended to do further test in a WHO reference laboratory.^65^ In addition to ORF1ab and N genes, the detection of S gene is also considered by many laboratories.

Nucleic acid detection technology has the characteristics of early diagnosis, high sensitivity and specificity. ‘Diagnosis and Treatment Guideline of the Novel Coronavirus Pneumonia’ of China proposed to use fluorescent‐tagged probe based real‐time RT‐PCR to detect SARS‐CoV‐2 nucleic acid in respiratory specimens or blood specimens (Table 1).

**Table 1:** The changes of nucleic acid test and serological test in the Diagnosis and Treatment Guideline of COVID‐19 from version 2 to 7 in China

Description	Ver 7 (Mar 4)	Ver 6 (Feb 18)	Ver 5 (Feb 4)	Ver 4 (Jan 27)	Ver 3 (Jan 23)	Ver 2 (Jan 18)
Laboratory test	*1. Aetiological test: nucleic acid test in the specimens of pharyngeal swab, sputum and other lower respiratory tract secretions, blood, faeces by real‐time RT‐PCR and NGS.* *Sputum and lower respiratory tract secretions are more accurate;* *2. Serological test: novel coronavirus IgM antibody presents on the 3‐5 days after COVID‐19 onset, IgG antibody titre increases 4 times or more in recovery phase compared to acute phase.*	Nucleic acid test in the specimens of pharyngeal swab, sputum and other lower respiratory tract secretions, blood. *To make sure the accurate result, recommend taking the sputum and secretions from the lower respiration track by tracheal* intubation.	Nucleic acid test in the specimens of pharyngeal swab, sputum and other lower respiratory tract secretions, blood and faeces.	Nucleic acid test *in the specimens of pharyngeal swab, sputum and other lower respiratory tract secretions, blood.*	NA	NA
Diagnosis confirmation	The suspected case, who have one of the following aetiological evidences: 1.Nucleic acid test is positive by real‐time RT‐PCR. 2.Have high identity with the known novel coronavirus sequence by NGS. *3.Serological test: both the specific IgM and IgG antibody of SARS‐CoV‐2 are positive; serological IgG antibody becomes positive or IgG antibody titre increases 4 times or more in recovery phase compared to acute phase*	The suspected case, who have one of the following aetiological evidences: 1.Nucleic acid test is positive by real‐time RT‐PCR 2.Have high identity with the known novel coronavirus sequence by NGS	The suspected case, who have one of the following aetiological evidences: 1. Nucleic acid test is positive by real‐time RT‐PCR in the respiratory or blood specimens. 2. Have high identity with the known novel coronavirus sequence by NGS in the respiratory or blood specimens	The suspected case, who have one of the following aetiological evidences: 1. Nucleic acid test is positive by real‐time RT‐PCR *in the respiratory or blood specimens.* 2. Have high identity with the known novel coronavirus sequence by NGS *in the respiratory or blood specimens*	No change	Conform to the suspected case standard and have the following: 1.Nucleic acid test is positive by real‐time RT‐PCR in the specimens of sputum, pharyngeal swab and lower respiratory tract secretions. 2.Have high identity with the known novel coronavirus sequence by NGS in the specimens of sputum, pharyngeal swab and lower respiratory tract secretions
Report system	*The suspected case can be excluded if fitting for the following two conditions: 1. Nucleic acid test is negative in two consecutive times (the interval of sampling time is at least 24 h).* *2. And the specific IgM and IgG antibody of SARS‐CoV‐2 are negative after 7‐d onset*	The suspected cases should be isolated and tested for nucleic acid of SARS‐CoV‐2	No change	No change	No change	The suspected cases should be isolated and introduced for multidiscipline diagnosis within 2h; If necessary, they should be tested for nucleic acid of SARS‐CoV‐2. The suspected case can be excluded if nucleic acid test is negative in two consecutive times (the interval of sampling time is at least 1 day).
Release from quarantine	*The out‐of‐hospital standard: Nucleic acid test is negative in two consecutive times (the interval of sampling time is at least 24 h) in the specimens of sputum and respiratory tract secretion etc*	No change	No change	No change	No change	The out‐of‐hospital standard: Nucleic acid test is negative in two consecutive times (the interval of sampling time is at least 1 d) in the respiratory tract specimens.

Note

The italicized text indicates the increased contents in the guideline compared with the previous version.

Although the viral nucleic acid test is currently the standard method for diagnosis of COVID‐19, the obvious limitations restrict its extensive use: (a) it usually takes 2‐3 hours to produce results; (b) real‐time PCR test requires certified laboratories, expensive equipment and trained technicians for operation; and (c) false negatives exist in real‐time RT‐PCR of COVID‐19.^66^ Recently, Abbott ID NOW molecular diagnostic platform technology developed ultra‐fast DNA or RNA amplification, combined with fluorescence detection system based on the nicking endonuclease amplification reaction (NEAR) technology, which could get bacterial or virus test results within 13 minutes or less (positive results within 5 minutes, negative results within 15 minutes). This technology will substantially speed up the population screening and COVID‐19 patient identification.^67^

### 7.2 Antibody detection

Rapid and accurate detection of SARS‐CoV‐2‐specific antibodies in the blood of patients is one of the options for supplemental nucleic acid testing. IgM provides the first line of defence during viral infection, followed by the production of IgG that is important for long‐term immunity and immunological memory.^68^ Moreover, positive IgM antibody often indicates an acute phase of infection while positive IgG antibody indicates late or previous infection. Therefore, we believe that detection of IgM and IgG can also provide information on the time course of virus infection. However, since serological test was gradually established, its diagnostic function was only recognized in ‘Diagnosis and Treatment Guideline of the Novel Coronavirus Pneumonia (Version 7)’ of China (Table 1).

According to a recent study, an increase of viral antibodies can be seen in almost all patients on day 5 after COVID‐19 onset and the positive rates of IgM and IgG were 81% and 100%, respectively.^69^ Therefore, dynamic monitoring of viral IgM or IgG can be considered as a complementary way for nucleic acid detection and could also help to diagnose the COVID‐19 patient with negative result of nucleic acid test but positive antibody.^70^ In addition, antibody test could be crucial to inform public policymakers how many asymptomatic cases have occurred in a population^71^ since it is the most convenient and fast approach to identify the asymptomatic cases so far.

## 8 TREATMENT

Based on the previously therapeutic experience of SARS and MERS, the potential treatments for COVID‐19 include antiviral drugs, plasma transfusion, vaccines and so on. Given the rapid outbreak and spread of COVID‐19, repurposing the existing antiviral drugs such as anti‐HIV drugs, anti‐HBV and anti‐HCV drugs is the fast and crucial experimentally therapeutic options and the clinical efficacy of these drugs requires clinical trials to prove.^72^ Recently, the WHO director general Tedros Adhanom has announced that WHO will lead clinical trials of four candidate drugs (remdesivir, lopinavir/ritonavir, interferon β and chloroquine) against COVID‐19 alone or in combination and more than 70 countries have joined WHO's Solidarity Trial to accelerate the search for an effective treatment.^73^ So far, a total of 1119 clinical trials have been registered to evaluate therapeutics for COVID‐19 as of 18 April 2020 (https://www.who.int/blueprint/priority‐diseases/key‐action/novel‐coronavirus/en/). In addition, stem cells are also expected to inhibit the overactivation of immune system, control acute pulmonary inflammation and improve the endogenous reparation for injured tissue,^74^, ^75^ which will become a new exploration in the treatment of severe COVID‐19. It also brings new hope for the treatment of novel coronavirus infection‐related acute respiratory distress syndrome. A clinical trial (NCT04252118) of COVID‐19 stem cell therapy sponsored by Beijing 302 Hospital is under way.

### 8.1 Antiviral drugs

Antiviral drugs can be classified as viral protease inhibitors, viral nucleoside analogs and agents that disrupt the interaction mechanism between the virus and the host (Table 2).

**Table 2:** Potential antiviral drugs against COVID‐19

Drug	Antiviral mechanism	References	Evidences	Therapeutic effect
Lopinavir/Ritonavir	Viral protease inhibitors	Kim et al 2020	Clinical case study	Did not decease the viral load and have gastrointestinal side effect
		Cao et al 2020	Clinical trial	No benefit was observed in hospitalized adult patients with severe Covid‐19
Disulfiram		Li and De Clercq 2020	In vitro	Has inhibitory activity against SARS and MERS
Favipiravir	Viral nucleoside analogs	Wang et al 2020	In vitro	Favipiravir could suppress SARS‐COV‐2 infection at a high concentration
Remdesivir		Holshue et al 2020	Clinical case study	Cure a COVID‐19 patient in the United States by intravenous injection
		Wang et al 2020	In vitro	Remdesivir could block SARS‐CoV‐2 enter Vero E6 cells
		Gao et al 2020	In vitro	Revealed the possible inhibition mechanism of SARS‐COV‐2 RNA polymerase by Remdesivir effector molecules
		Grein et al 2020	Clinical trial	Remdesivir is effective in treating severe COVID‐19.
Ribavirin and Galidesivir		Li and De Clercq 2020	No evidence	Have therapeutic effect on patients with SARS and MERS but lacking the evidence for SARS‐COV‐2
Chloroquine	Virus‐host fusion inhibitors	Wang et al 2020	In vitro	Suppression of COVID‐19 pneumonia exacerbation, reduction of symptom duration,
		Gao et al 2020	Clinical trial	Have apparent efficacy and acceptable safety against COVID‐19‐associated pneumonia in multicentre clinical trials conducted in China.
Hydroxychloroquine		Wang et al 2020	In vitro	Have the same antiviral mechanism with chloroquine
		Gautret et al 2020	Clinical trial	Hydroxychloroquine treatment is significantly associated with viral load reduction/disappearance in COVID‐19 patients and its effect is reinforced by azithromycin.
Arbidol		Dong et al 2020	In vitro	Arbidol can effectively inhibit SARS‐CoV‐2 infection at a concentration of 10‐30 µM
Nitazoxanide		Wang et al 2020	In vitro	Have inhibition activity of the SARS‐COV‐2
IDX‐184		Elfiky 2020	In vitro	Have effective against SARS‐CoV‐2 strain.
Sofosbuvir		Elfiky 2020	In vitro	Tightly combine with SARS‐CoV‐2 RdRp for viral clearance
Corticosteroids		Zhou and Liu 2020	Clinical experience	Low‐dose corticosteroids may be beneficial for the survival of severe COVID‐19 patients
Baricitinib	JAK signalling pathway inhibitor	Richardson and Griffin 2020	Benevolent AI‐derived knowledge graph	Baricitinib could also abate SARS‐COV‐2 virus infectivity
Traditional Chinese medicine	Antiviral, anti‐immune/inflammatory and hypoxic reactions	Zhang et al 2020	In vitro evidence and clinical experience	May also directly inhibit the SARS‐CoV‐2

### 8.2 Viral protease inhibitors

Lopinavir/ritonavir and disulfiram are the well‐known viral protease inhibitors and have been shown to be effective against SARS and MERS.^76^ Lopinavir/ritonavir were reported to reduce mortality, intubation rate and the use of methylprednisolone when introduced as a treatment of SARS patients in early stage.^76^, ^77^ A recent study reported two COVID‐19 cases in South Korea and found that they did not have a significant decrease of the viral load after lopinavir/ritonavir therapy, but experienced gastrointestinal discomfort as a side effect.^78^ Recent published result of the first clinical trial for lopinavir/ritonavir indicated that no benefit effect was observed after lopinavir/ritonavir treatment beyond standard care in hospitalized severe COVID‐19 patients.^79^ Disulfiram is a drug for the treatment of alcohol dependence, and studies have confirmed its inhibitory activity against SARS and MERS in cell cultures instead of clinical situation.^72^

### 8.3 Viral nucleoside analogs

Favipiravir has shown an effective inhibition on Ebola, yellow fever, chikungunya, norovirus and enterovirus by restraining RNA‐dependent RNA polymerase (RdRp) of RNA viruses.^80^ A recent study demonstrated that Favipiravir could suppress SARS‐CoV‐2 infection at a high concentration (half‐maximal effective concentration (EC50) = 61.88 μmol/L, half‐cytotoxic concentration (CC50) >400 μmol/L, selectivity index (SI)> 6.46).^81^ Remdesivir, approved for HIV infection treatment, has been used to cure a COVID‐19 patient in the United States by intravenous injection in January 2020.^42^ A recent in vitro experiment displayed that remdesivir could block SARS‐CoV‐2 to enter Vero E6 cells (ATCC‐1586) at low concentrations.^81^ To determine the clinical efficacy of Remdesivir, two Phase III clinical trials are underway (NCT04252664 and NCT04257656).^72^, ^82^ The efficacy of nucleotide analogs requires viral RdRps to recognize and successfully incorporate the active form of the inhibitors into the growing RNA strand, which is the possible inhibition mechanism of SARS‐CoV‐2 RNA polymerase by remdesivir effector molecules.^83^ The latest clinical trial studied the effect of compassionate use of remdesivir in severe COVID‐19 patients, and clinical improvement was observed in 36 of 53 patients (68%), which indicated that remdesivir was effective in treating severe COVID‐19.^84^

### 8.4 Virus‐host fusion inhibitors

In a study involving more than 100 patients, chloroquine was superior to the control group in suppression of COVID‐19 pneumonia exacerbation, reduction of symptom duration and delay of viral clearance without severe side effects,^85^ but no specific primary or secondary end‐points. For clinicians accustomed to it, hydroxychloroquine may be preferable for clinical SARS‐CoV‐2 treatment. To obtain a better therapeutic effect, a loading dose is recommended prior to a maintenance dose.^81^, ^86^, ^87^ A recent small‐scale study of COVID‐19 found that most patients taking chloroquine cleared the novel coronavirus much faster than the control group and adding azithromycin to the medicine could significantly improve the efficiency of eliminating viruses.^88^ However, a recent study indicated that chloroquine and hydroxychloroquine could not only be useless in treating COVID‐19 patients but even harmful. Hence, nevertheless the proved in vitro efficacy, before clinical trials results publication and/or further clarification about COVID‐19 pathogenesis, clinicians should use it cautionally.^88^, ^89^, ^90^ At present, high quality, adequately powered randomized clinical trials in primary and secondary care settings are urgently required to guide policymakers and clinicians. These studies should report medium‐ and long‐term follow‐up results and safety data.^90^ Arbidol is an anti‐influenza drug and also used in anti‐HCV treatment. An in vitro study has revealed that Arbidol could effectively inhibit SARS‐CoV‐2 infection at a concentration of 10‐30 µmol/L, which brought arbidol the potential to treat COVID‐19 clinically.^82^ Nitazoxanide, as an anti‐diarrhoea medicine, has been shown inhibition activity of the SARS‐CoV‐2 in in vitro studies.^81^ In the future, combining the molecular mechanisms of these two agents could develop more effective compounds to prevent emerging infections.^91^

### 8.5 Others

Researches indicated that appropriate use of low‐dose corticosteroids may be beneficial for the survival of severe COVID‐19 patients; however, it should strictly comply with the guidelines for recommended COVID‐19 patients, such as patients with refractory ARDS, sepsis or septic shock.^91^, ^92^ Based on benevolent AI's proprietary artificial intelligence (AI)‐derived knowledge graph, baricitinib could also abate SARS‐CoV‐2 virus infectivity, virus replication and abnormal host inflammatory response in combination with direct antiviral drugs (lopinavir or ritonavir and remdesivir). This approach could facilitate the subsequent development of anti‐SARS‐CoV‐2 agents.^93^ The mechanism of Chinese herbal medicines against COVID‐19 may include the following process, such as antiviral, immune/inflammatory, hypoxic reactions and so on, and it may also directly inhibit the SARS‐CoV‐2.^94^

### 8.6 Convalescent plasma therapy

Convalescent plasma or immunoglobulins have been shown to improve the survival rate of SARS patients with progressive deterioration.^95^ In the pandemic of 2009 influenza A H1N1 (H1N1pdm09) infection, convalescent plasma therapy was found to help to reduce the relative risk of mortality significantly.^96^ During the outbreak of Ebola virus disease (EVD) in 2014, the use of convalescent plasma collected from EVD recovery patients was recommended by WHO as one of the treatment modalities.^97^ In 2015, the laboratory effect of convalescent plasma therapy for patients with Middle East respiratory syndrome coronavirus infection was reported^98^ and no severe adverse events occurred among those patients cured by convalescent plasma. An in vivo study also showed that the effects of this antibody were not only limited to free viral clearance and block new infection, but also could accelerate the clearance of infected cells.^99^

Currently, it was suggested to collect whole blood or plasma from recovery patients for transfusion to the progressive rapidly, severe and critical COVID‐19 patients during the SARS‐COV‐2 outbreak, but the following principles should be followed: (a) the patient's disease course should not exceed 3 weeks or has viraemia and (b) patients with rapid progression and early severity should be evaluated comprehensively by clinical experts.^100^ A recent study showed that conditions of five COVID‐19 patients have been improved after treating with the plasma of the rehabilitated patients.^101^ Two of these antibodies were tested and showed strong anti‐SARS‐CoV‐2 capabilities by the reduction of the binding of SARS‐CoV‐2 S protein RBD to human ACE2 with reduction rate of 99.2% and 98.5%, respectively.^102^ However, more clinical research should be conducted to prove the safety and effectiveness of convalescent plasma therapy among COVID‐19 patients.^103^

### 8.7 Vaccine

Genomic sequence studies displayed that the spike protein of SARS‐CoV‐2 has high identity with that of SARS and MERS, which might indicate the similarity of immune evasion mechanism among SARS‐CoV‐2, SARS and MERS.^104^, ^105^ On the basis of knowledges obtained from SARS and MERS vaccine development, several research groups have announced to start SARS‐CoV‐2 vaccine R&D immediately after the global outbreak.^106^, ^107^ As was reported, receptor‐binding domain in full‐length spike (S) or S1 was crucial for SARS‐CoV‐2 entry into the host cell.^25^, ^107^ This protein is a good vaccine antigen‐inducing neutralizing antibodies to prevent host cell attachment and against coronavirus infection.^108^, ^109^ Live attenuated vaccines and inactivated vaccines are based on whole virion.^37^, ^110^ During Zika virus outbreak, DNA vaccine was the first vaccine candidate that entered clinical trial.^111^, ^112^, ^113^ So far, WHO has listed the detailed information of 86 vaccines under development worldwide, including DNA, RNA, adenoviral vector and recombinant protein vaccine, and six of them have entered into clinical trial (Supplementary Table). To develop SARS‐CoV‐2 vaccine successfully, important information related to vaccine development and evaluation should be well defined, including targeted antigen(s), immunization route, related immune protection, animal models, outbreak forecasting and targeted population.^113^, ^114^ However, the production process and preclinical information of vaccines should be assessed to ensure volunteers’ safety prior to clinical testing.^114^ In a recent interview of the Harvard Office of Technology Development (OTD), researchers suggested that further research and development on a class of molecules called bisphosphonates might turbocharge a vaccine against SARS‐CoV‐2 and help bring immunity to huge populations more quickly.^115^

## 9 CONCLUSION

The pandemic of COVID‐19 has lasted for around 5 months, and so far, the main affected areas have from the original outbreak China to outside regions, especially American and Europe. Fortunately, we already accumulated some knowledge about the pathogen virus, its epidemiological and clinical features, pathogenesis and some effective therapeutic approaches, summarized above, which have contributed to the success against COVID‐19 and will help human being completely control the epidemic finally.

## CONFLICTS OF INTEREST

The authors declare no conflict of interest.

## AUTHOR CONTRIBUTIONS

HL and ZL collected, analysed and interpreted the data. HL and J. G. conceived and supervised the study. HL wrote the manuscript. All authors read and approved the final manuscript.

## Supporting information

Table S1

## Data Availability

All data, models and code generated or used during the study appear in the submitted article.
