# Supplementary material for: Scientific research progress of COVID‐19/SARS‐CoV‐2 in the first five months
Source: J Cell Mol Med. 2020 May 7;24(12):6558–70. doi: 10.1111/jcmm.15364 (PMC7264656; doi:10.1111/jcmm.15364)
Supplement: Supplementary file 1 — Table S1 [file JCMM-24-6558-s001.docx]

| **Supplementary Table. COVID-19 vaccines under development** | | | | | | | | | |
| --- | --- | --- | --- | --- | --- | --- | --- | --- | --- |
| **Number** | **Type of Vaccine** | **Related Use/Platform** | **Developer/Researcher** | **Current Stage of Development** | **Funding Sources** | **Clinical Trials for COVID-19** | **Anticipated Next Steps Timing** | **Published Results** | **Sources** |
| 1 | DNA plasmid; INO-4800 | Same platform as vaccine candidates for Lassa, Nipah, HIV, Filovirus, HPV, cancer indications, Zika, and Hepatitis B | Inovio Pharmaceuticals/Beijing Advaccine Biotechnology | Clinical | Coalition for Epidemic Preparedness (CEPI)/Gates Foundation | NCT04336410  (Inovio  Pharmaceuticals) | Started Phase 1 April 2020; initial data expected late summer 2020 | | World Health Organization MarketWatch BioAegis Therapeutics INOVIO |
| 2 | DNA |  | Takis/Applied DNA Sciences/Evvivax | Pre-clinical |  |  | Prelinical results expected in April 2020; Phase 1 to start in fall 2020 | | World Health Organization Takis |
| 3 | DNA plasmid |  | OPENCORONA -  Cobra Biologics/Karolinska Institute | Pre-clinical | European Commission (Horizon 2020 Program) | | Phase 1 to start in 2020 |  | BioSpace |
| 4 | DNA plasmid |  | Osaka University/AnGes/Takara Bio | Pre-clinical |  |  |  |  | World Health Organization |
| 5 | DNA plasmid |  | Zydus Cadila | Pre-clinical |  |  |  |  | World Health Organization |
| 6* | DNA plasmid, needle-free delivery* | Same platform as vaccine candidates for SARS* | Immunomic Therapeutics/EpiVax/ PharmaJet* | Pre-clinical* |  |  |  |  | World Health Organization* |
| 7* | Inactivated (formaldehyde- inactivated + alum) | Same platform as vaccine candidates for SARS | Sinovac | Pre-clinical |  |  |  |  | World Health Organization |
| 8* | Inactivated |  | Beijing Institute of Biological Products/Wuhan Institute of Biological Products | Pre-clinical |  |  |  |  | World Health Organization |
| 9* | Inactivated |  | Osaka University/BIKEN/NIBIOHN | Pre-clinical |  |  |  |  | World Health Organization |
| 10* | Deoptimized live attenuated virus | Same platform as vaccine candidates for HAV, InfA, ZIKV, FMD, SIV, RSV, DENV | Codagenix/Serum Institute of India | Pre-clinical |  |  | Animal data in summer 2020 | | World Health Organization Indian Express |
| 11* | Replicating viral vector, influenza vector expressing RBD | Same platform as vaccine candidates for MERS | The University of Hong Kong | Pre-clinical | Coalition for Epidemic Preparedness (CEPI) | |  |  | World Health Organization Coalition for Epidemic Preparedness World Health Organization |
| 12* | CoroFlu, self-limiting influenza virus | Same platform as vaccine candidates for influenza | University of Wisconsin-Madison / FluGen/ Bharat Biotech | Pre-clinical |  |  | Start Phase 1 trial in fall 2020 | | Press release from the  collaboration |
| 13* | Non-replicating viral vector; MVA encoded VLP | Same platform as vaccine candidates for LASV, EBOV, MARV, HIV | GeoVax/BravoVax | Pre-clinical |  |  |  |  | World Health Organization GeoVax GeoVax |
| 14* | Non-replicating viral vector; Ad26 (alone or with MVA boost) | Same platform as vaccine candidates for Ebola, HIV, RSV | Janssen Pharmaceutical Companies/ Beth Israel Deaconess Medical Center | Pre-clinical | Biomedical Advanced Research and Development Authority (BARDA) | | Start Phase 1 in September 2020 | | World Health Organization Johnson & Johnson Johnson & Johnson FierceBiotech Johnson & Johnson press  release |
| 15* | Non-replicating viral vector; ChAdOx1 | Same platform as vaccine candidates for influenza, TB, Chikungunya, Zika, MenB, plague | Consortium of the Jenner Institute, Oxford Biomedica, University of Oxford, Vaccines Manufacturing and Innovation Centre, Pall Life Sciences, Cobra Biologics, and HalixBV | Clinical* | Coalition for Epidemic Preparedness (CEPI)/UK Government | NCT04324606 (University of Oxford) | Animal trials begin March 2020, Phase 1 begins April 2020 | | World Health Organization Guardian Fierce Biotech World Health Organization PharmaTimes |
| 16* | Non-replicating viral vector | Same platform as vaccine candidates for many pathogens | DZIF - German Center for Infection Research | Pre-clinical |  |  |  |  | World Health Organization |
| 17* | AdCOVID; single-dose, intranasal vaccine; non replicating viral vector; adenovirus-based NasoVAX expressing spike protein | Same platform as vaccine candidates for influenza | Altimmune/University of Alabama at Birmingham | Pre-clinical |  |  | Phase 1 trial to begin Q3 2020 | | World Health Organization Altimmune press release Altimmune |
| 18* | Non-replicating viral vector; Ad5 S (GREVAX™ platform) | Same platform as vaccine candidates for MERS | Greffex | Pre-clinical |  |  |  |  | World Health Organization Greffex |
| 19* | Non-replicating viral vector; Oral Vaccine platform | Same platform as vaccine candidates for InfA, CHIKV, LASV, NORV, EBOV, RVF, HBV, VEE | Vaxart/Emergent BioSolutions | Pre-clinical |  |  | Phase 1 trial to begin in the second half of 2020 | | World Health Organization Vaxart press release Emergent BioSolutions |
| 20* | Non-replicating viral vector, MVA expressing structural proteins* | Same platform as vaccine candidates for HIV, HCV, CHIKV, EBOV, Zika, Malaria, Leishmania* | Centro Nacional Biotecnologia (CNB- CSIC), Spain* | Pre-clinical* |  |  |  |  | World Health Organization* |
| 21* | Non-replicating viral vector; Adenovirus Type 5 vector (Ad5- nCoV) | Same platform as vaccine candidates for EBOV | CanSino Biologics/Beijing Institute of Biotechnology | Clinical |  | NCT04313127 (CanSino  Biologics Inc.) | Phase 1 ends December 2020; Phase 2 to begin in April/May 2020 | ChiCTR2000030906  (Insitute of Biotechnology,  Academy of Military Medical Sciences, PLA of China) (Phase 1)* ChiCTR2000031781  (Insitute of Biotechnology,  Academy of Military Medical Sciences, PLA of China) (Phase 2)* | World Health Organization FiercePharma CanSino Biologics  announcement |
| 22* | Protein subunit, capsid-lide particle (CLP) | Same platform as vaccine candidates for HPV | PREVENT-nCoV consortium (AdaptVac, Institute for Tropical Medicine at University of Tubingen, Leiden University Medical Center, University of Copenhagen, ExpreS2ion Biotechnologies, Wageningen University) | Pre-clinical | European Commission (Horizon 2020 Program) | | Phase 1 to begin by February 2021 | | AdaptVac  World Health Organization ExpreS2ion press release |
| 23* | Protein subunit, drosophila S2 insect cell expression system VLPs* | | ExpreS2ion* | Pre-clinical* |  |  |  |  | World Health Organization* |
| 24* | Protein subunit; S protein |  | WRAIR/USAMRIID | Pre-clinical |  |  |  |  | World Health Organization |
| 25* | Protein subunit, S protein + adjuvant | Same platform as vaccine candidates for Influenza | National Institute of Infectious Disease, Japan | Pre-clinical |  |  |  |  | World Health Organization |
| 26* | Protein subunit, VLP- recombinant protein + adjuvant | | Osaka University/BIKEN/National Institutes of Biomedical Innovation, Japan | Pre-clinical |  |  |  |  | World Health Organization |
| 27* | Protein subunit, native like trimeric subunit spike protein | Same platform as vaccine candidates for HIV, RSV, Influenza | Clover Biopharmaceuticals Inc./GSK/ Dynavax | Pre-clinical |  |  |  |  | World Health Organization World Health Organization |
| 28* | Protein subunit; peptide |  | Vaxil Bio | Pre-clinical |  |  |  |  | World Health Organization |
| 29* | Protein subunit; adjuvanted protein subunit (RBD) | | Biological E Ltd | Pre-clinical |  |  |  |  | World Health Organization |
| 30* | Protein subunit; S protein |  | AJ Vaccines | Pre-clinical |  |  |  |  | World Health Organization |
| 31* | Protein subunit; S protein |  | Vaxine Pty Ltd/ Flinders University / Oracle | Pre-clinical |  |  |  |  | Flinders University press release |
| 32* | Protein subunit; Ii-Key peptide | Same platform as vaccine candidates for HIV, SARS-CoV, Influenza | Generex/EpiVax | Pre-clinical |  |  |  |  | World Health Organization Generex press release EpiVax |
| 33* | Protein subunit; S protein | Same platform as vaccine candidates for Inf H7N9 | EpiVax/University of Georgia | Pre-clinical |  |  |  |  | World Health Organization EpiVax |
| 34* | PittCoVacc, Protein subunit, microneedle arrays S1 subunit | Same platform as vaccine candidates for MERS | University of Pittsburgh | Pre-clinical |  |  | Phase 1 to start as early as June 2020 | | University of Pittsburgh press release EBioMedicine World Health Organization |
| 35* | Protein subunit; S protein | Influenza, Ebola | University of Cambridge/DIOSynVax | Pre-clinical |  |  | Phase 1 to start as early as June 2020 | | University of Cambridge |
| 36* | Protein subunit; COVID-19 XWG-03 truncated Spike proteins | Same platform as vaccine candidates for HPV | Innovax/Xiamen University/GSK | Pre-clinical |  |  |  |  | FierceBiotech World Health Organization |
| 37* | Protein subunit; S protein, baculovirus production | Same platform as vaccine candidates for Influenza, SARS-CoV (FDA-approved vaccine) | Sanofi Pasteur/GSK* | Pre-clinical | Biomedical Advanced Research and Development Authority (BARDA) | | Start Phase 1 in second half of 2020* | | World Health Organization Sanofi Stat News MarketWatch Sanofi* |
| 38* | NVX-CoV2373; Protein subunit; Full length S trimers/nanoparticle + Matrix M* | Same platform as vaccine candidates for RSV, CCHF, HPV, VZV, EBOV | Novavax/Emergent BioSolutions | Pre-clinical | Coalition for Epidemic Preparedness (CEPI) | | Start Phase 1 in May 2020* | | World Health Organization Emergent BioSolutions Endpoints News* |
| 39* | Protein subunit (gp-96 backbone) | Same platform as vaccine candidates for cancer (NSCLC), HIV, malaria, Zika | Heat Biologics (Zolovax) /University of Miami | Pre-clinical |  |  |  |  | World Health Organization Clinical Trials Arena Heat Biologics |
| 40* | Protein subunit; molecular clamp stabilized Spike protein | Same platform as vaccine candidates for Nipah, influenza, Ebola, Lassa | University of Queensland/GSK/ Dynavax | Pre-clinical | Coalition for Epidemic Preparedness (CEPI)/ Queensland Government/ Federal Government (Australia)/Paul Ramsay Foundation | | | | World Health Organization ABC News Australia Dynavax World Health Organization |
| 41* | Protein subunit; S1 or RBD protein | Same platform as vaccine candidates for SARS | Baylor College of Medicine | Pre-clinical |  |  |  |  | World Health Organization |
| 42* | Protein subunit; Subunit protein, plant produced | | iBio/CC-Pharming | Pre-clinical |  |  |  |  | World Health Organization |
| 43* | Protein subunit, recombinant protein, nanoparticles (based on S-protein and other epitopes) | | St. Petersburg Scientific Research Institute of Vaccines and Serums | Pre-clinical |  |  |  |  | World Health Organization |
| 44* | Protein subunit, adjuvanted microsphere peptide | | VIDO-InterVac, University of Saskatchewan | Pre-clinical | The Government of Saskatchewan and the Canadian Federal Government | | Animal testing results expected in April 2020 | | World Health Organization World Health Organization Government of Saskatchewan |
| 45* | Protein subunit, peptide | Same platform as vaccine candidates for Ebola, Marburg, HIV, Zika, Influenza, HPV therapeutic vaccine, Breast Cancer | Flow Pharma | Pre-clinical |  |  |  |  | World Health Organization |
| 46* | Protein subunit, synthetic long peptide vaccine candidate for S and M proteins | | OncoGen | Pre-clinical |  |  |  |  | World Health Organization |
| 47* | DPX-COVID-19, protein subunit, lipid-based delivery platform | Same platform as vaccine candidates for cancer and infectious diseases, including malaria and anthrax | IMV, Inc. / Canadian Center for Vaccinology at Dalhousie University/ Izaak Walton Killam Health Center/ Nova Scotia Health Authority; Canadian Immunization Research Network / University of Laval / Global Urgent and Advanced Research and Development in Canada | Pre-clinical |  |  | Start Phase 1 testing by summer 2020 | | IMV, Inc. IMV, Inc. |
| 48* | Pan-coronavirus vaccine candidate, targeting COVID-19, SARS, and MERS, spike protein | | VBI Vaccines / National Research Council of Canada | Pre-clinical |  |  | Start Phase 1 testing by end of 2020 | | VBI Vaccines press release |
| 49* | Replicating viral vector; measles vector | | Zydus Cadila | Pre-clinical |  |  |  |  | World Health Organization |
| 50* | Replicating viral vector; measles vector | Same platform as vaccine candidates for West Nile, CHIKV, Ebola, Lassa, Zika, MERS | Institut Pasteur/Themis/University of Pittsburgh | Pre-clinical | Coalition for Epidemic Preparedness (CEPI) | | Start animal testing in April 2020 | | World Health Organization University of Pittsburgh Medical Center Coalition for Epidemic Preparedness |
| 51* | Live attenuated virus, measles virus | Same platform as vaccine candidates for Zika, H7N9, CHIKV | DZIF - German Center for Infection Research | Pre-clinical |  |  |  |  | World Health Organization |
| 52* | Replicating viral vector; horsepox vector; TNX-1800 | Same platform as vaccine candidates for smallpox, monkeypox | Tonix Pharma/Southern Research | Pre-clinical |  |  |  |  | World Health Organization Tonix Pharmaceuticals press release |
| 53* | Replicating viral vector, live viral vectored vaccine based on attenuated influenza virus backbone (intranasal)* | | BIOCAD/IEM* | Pre-clinical* |  |  |  |  | World Health Organization* |
| 54* | Replicating viral vector, VSV vector expressing S protein | Same platform as vaccine candidates for Ebola, Marburg, Lassa | IAVI/ Batavia | Pre-clinical |  |  |  |  | World Health Organization |
| 55* | RNA; LNP-encapsulated mRNA cocktail encoding VLP | | Fudan University/Shanghai JiaoTong University/RNACure Biopharma | Pre-clinical |  |  |  |  | World Health Organization |
| 56* | RNA; LNP-encapsulated mRNA cocktail encoding RBD | | Fudan University/Shanghai JiaoTong University/RNACure Biopharma | Pre-clinical |  |  |  |  | World Health Organization |
| 57* | RNA; Replicating defective SARS-CoV-2 derived RNAs* | | Centro Nacional Biotecnologia (CNB- CSIC), Spain* | Pre-clinical* |  |  |  |  | World Health Organization* |
| 58* | RNA; LNP-encapsulated mRNA | Same platform as vaccine candidates for MERS | University of Tokyo/ Daiichi-Sankyo | Pre-clinical |  |  |  |  | World Health Organization |
| 59* | RNA; mRNA |  | China CDC/Tongji University/Stermina | Pre-clinical |  |  |  |  | World Health Organization |
| 60* | RNA; LNP-encapsulated mRNA (mRNA 1273) | Same platform as vaccine candidates for multiple candidates | Moderna/NIAID | Clinical | Coalition for Epidemic Preparedness (CEPI)/ Biomedical Advanced Research and Development Authority (BARDA) | NCT04283461 (National Institute of Allergy and Infectious Diseases) | Phase 1 started March 2020, study ends June 2021 | | World Health Organization Wall Street Journal MarketWatch ClinicalTrials.gov |
| 61* | LUNAR-COV19; RNA; mRNA* | Same platform as vaccine candidates for multiple candidates | Arcturus/Duke-NUS | Pre-clinical |  |  | Start Phase 1 in summer 2020* | | World Health Organization Arcturus Therapeutics Arcturus Therapeutics* |
| 62* | RNA; saRNA | Same platform as vaccine candidates for EBOV, LASV, MARV, Inf (H7N9), RABV | Imperial College London | Pre-clinical |  |  | Start Phase 1 in summer 2020 | | World Health Organization Imperial College London |
| 63* | RNA; mRNA | Same platform as vaccine candidates for RABV, LASV, YFV, MERS, InfA, ZIKV, DengV, NIPV | CureVac | Pre-clinical | Coalition for Epidemic Preparedness (CEPI); European Commission | | Start Phase 1 in June 2020 | | World Health Organization Labiotech.eu |
| 64* | RNA; BNT162 |  | BioNTech/Fosun Pharma/Pfizer | Pre-clinical |  |  | Start Phase 1 late April 2020 | | FierceBiotech Endpoints News World Health Organization |
| 65* | RNA; liposome-encapsulated mRNA* | Same platform as vaccine candidates for cancer | BIOCAD | Pre-clinical |  |  | Animal studies begin in April 2020 | | BIOCAD |
| 66* | RNA; mRNA |  | Sanofi Pasteur/ Translate Bio | Pre-clinical |  |  |  |  | Translate Bio |
| 67* | RNA; mRNA (cross-strain protective COV-2 mRNA) vaccine for high-risk populations | | eTheRNA Immunotherapies/EpiVax/ Nexelis/REPROCELL/Centre for the Evaluation of Vaccination of the University of Antwerp | Pre-clinical |  |  | Start Phase 1 early 2021 |  | EpiVax |
| 68* | RNA |  | GeneOne Life Science/Houston Methodist | Pre-clinical |  |  |  |  | Houston Methodist |
| 69* | VLP; virus-like particle, based on RBD displayed on virus-like particle* | | Saiba GmbH* | Pre-clinical* |  |  |  |  | World Health Organization* |
| 70* | VLP; plant-derived VLP | Same platform as vaccine candidates for flu, rotavirus, norovirus, West Nile virus, and cancer | Medicago Inc. | Pre-clinical |  |  | Start Phase 1 in July/ August 2020 | | World Health Organization Medicago press release |
| 71* | VLP; ADDomerTM multiepitope display | | Imophoron Ltd/Bristol University’s Max Planck Centre | Pre-clinical |  |  |  |  | World Health Organization University of Bristol |
| 72* | Gene-encoded antibody vaccine, non-viral nanoparticle delivery | | SmartPharm Therapeutics/Sorrento Therapeutics | Pre-clinical |  |  |  |  | SmartPharm Therapeutics |
| 73* | Self-assembling vaccine (fusion protein of a heat shock protein and Avidin, with biotinylated immunogenic peptides) | | HaloVax (Voltron Therapeutics)/The Vaccine & Immunotherapy Center at the Massachusetts General Hospital | Pre-clinical |  |  | Animal study results by October 2020 | | Voltron Therapeutics press release |
| 74* | LV-SMENP-DC Dendritic cells modified with lentiviral vector expressing synthetic minigene based on domains of selected viral proteins; administered with antigen-specific cytotoxic T lymphocytes | | Shenzhen Geno-Immune Medical Institute | Clinical* |  | NCT04276896 (Shenzhen Geno-Immune Medical  Institute) | | | Nature |
| 75* | Artificial antigen-presenting cells modified with lentiviral vector expressing synthetic minigene based on domains of selected viral proteins | | Shenzhen Geno-Immune Medical Institute | Clinical* |  | NCT04299724 (Shenzhen Geno-Immune Medical  Institute) | | | Nature |
| 76* | ISR-50 |  | ISR Immune System Regulation | Pre-clinical |  |  | Animal study results expected in Q2 2020, Phase 1 begins Q4 2020 | | ISR Immune System Regulation |
| 77* | Unknown |  | ImmunoPrecise | Pre-clinical |  |  |  |  | World Health Organization |
| 78* | Unknown |  | MIGAL Galilee Research Institute | Pre-clinical |  |  |  |  | World Health Organization |
| 79* | Unknown |  | Doherty Institute | Pre-clinical |  |  |  |  | World Health Organization |
| 80* | Unknown |  | Tulane University | Pre-clinical |  |  |  |  | World Health Organization Clinical Trials Arena |
| 81* | Unknown |  | SK Biosciences | Pre-clinical |  |  | Phase 1 begins as early as September 2020 | | UPI |
| 82* | Unknown |  | Vir Biotechnology/GSK | Pre-clinical |  |  |  |  | Vir Biotechnology |
| 83* | Unknown |  | Precision Vaccines Program at Boston Children’s Hospital | Pre-clinical |  |  |  |  | Scientific American |
| 84* | Unknown, tobacco plant technology |  | Kentucky BioProcessing (British American Tobacco) | Pre-clinical |  |  |  |  | BAT |
| 85* | Unknown |  | ReiThera | Pre-clinical |  |  |  |  | World Health Organization |
| 86* | Unknown |  | BioNet Asia | Pre-clinical |  |  |  |  | World Health Organization |
| **Abbreviation** | | CCHF = Crimean-Congo Haemorrhagic Fever; CHIKV = Chikungunya Virus; DengV = Dengue Virus; FMD = Foot and Mouth Disease; EBOV = Ebola Virus; HAV = Hepatitis A Virus; HBV = Hepatitis B Virus; HIV = Human Immunodeficiency Virus; HPV = Human Papilloma Virus; Inf = Influenza; LASV = Lassa Fever Virus; MARV = Marburg Virus; MenB = Mengingitis B; MERS = Middle East Respiratory Syndrome; NIPV = Nipah Virus; NORV = Norovirus; RABV = Rabies Virus; RSV = Respiratory Syncytial Virus; RVF = Rift Valley Fever; SARS = Severe Acute Respiratory Syndrome; SIV = Simian Immunodeficiency Virus; TB = Tuberculosis; VEE = Venezuelan Equine Encephalitis Virus; VZV = Varicella Vaccine (Chickenpox); YFV = Yellow Fever Virus; ZIKV = Zika Virus | | | | | | | |
| *** Indicates updated or new field; Updated April 15, 2020, at 2:30 p.m.** | | | | | | | | | |
| **Data sources from:**  1. World Health Organization (<https://www.who.int/blueprint/priority-diseases/key-action/novel-coronavirus/en/>)  2. Milken Institute (<https://milkeninstitute.org/covid-19-tracker>) | | | | | | | | | |
